# What’s new in HIV dermatology?

**DOI:** 10.12688/f1000research.16182.1

**Published:** 2019-06-28

**Authors:** Sarah J Coates, Kieron S Leslie

**Affiliations:** 1 Department of Dermatology, University of California San Francisco, 1701 Divisadero Street, San Francisco, CA, 94115, USA

**Keywords:** HIV, dermatology, nonmelanoma skin cancer, Kaposi sarcoma, Merkel cell carcinoma, syphilis, human papilloma virus, varicella zoster virus, herpes simplex virus, psoriasis, atopic dermatitis, prurigo nodularis, pruritus

## Abstract

HIV has long been associated with a number of inflammatory, infectious, and neoplastic skin conditions. In the era of anti-retroviral therapy, we have discovered even more about the relationship between skin disease and chronic immunosuppression. In particular, clinicians still face the propensity of persons living with HIV to develop difficult-to-control viral infections, chronic skin inflammation, and pruritus and—particularly as patients age—various types of skin cancers. Here, we summarize recent updates in the field of HIV dermatology and make recommendations to providers caring for these patients.

## Introduction

Since the early days of the human immunodeficiency virus (HIV) epidemic, dermatologists have confronted the myriad cutaneous manifestations that afflict persons living with HIV (PLWH). For years, the classic non-blanching violaceous plaques of Kaposi’s sarcoma (KS) were the most well-known outward signs of HIV infection, and other mucocutaneous findings—including warts, frequent herpes simplex virus outbreaks, and facial fat redistribution—foretold the status of patients’ immune systems and contributed to social stigmatization. Today, more than 20 years after the advent of highly active anti-retroviral therapy (HAART), we continue to discover more about the nuanced relationship between HIV infection and skin disease. As the number of PLWH increases by more than one million per year ^1^, understanding these dermatoses, recognizing what they communicate about the status of the immune system, and using effective management strategies are paramount.

## HIV-associated infectious dermatoses

### Human papilloma virus

Human papilloma virus (HPV) is one of the most prevalent infections among PLWH and its mucocutaneous manifestations—including both common and genital warts—can be extremely challenging to control. Mucocutaneous warts in PLWH may fail to respond to traditional treatments such as cryotherapy, topical imiquimod (Aldara), and topical podophyllin (Podocon), warranting more aggressive therapies such as intralesional cidofovir injections and rarely surgical intervention.

HPV co-infection accounts for the growing burden of anal high-grade squamous intraepithelial lesions (HSILs) and squamous cell carcinoma (SCC) among PLWH, particularly men who have sex with men (MSM) ^2^. HPV-related dysplastic changes may be found in up to 30% of individuals in this cohort; a recent study found that younger MSM with histories of inadequate viral suppression (prolonged time to diagnosis or inadequate treatment adherence or both) were at particularly high risk of developing HSIL ^2^. Physicians should consider HSIL or SCC when warts are not responding to treatment.

Widespread HPV vaccination may change the epidemiology of this infection in PLWH. Three preventative HPV vaccines have been developed: a bivalent vaccine against HPV-16 and -18 (Cervarix); a quadrivalent vaccine against HPV types 6, 11, 16, and 18 (Gardasil); and a nonavalent vaccine (Gardasil-9) that provides additional protection against types 31, 33, 45, 52, and 58 ^3^. All function by stimulating an antibody-mediated host response such that HPV is neutralized before it infects host cells ^3^. The Centers for Disease Control and Prevention (CDC) recommends HPV vaccination for all HIV patients who are 26 years old or younger ^4^. Clinical trials have demonstrated high vaccine immunogenicity (measured as antibody titer levels) in PLWH in this age range ^5^. Given that many diagnoses of HIV occur after age 26, it is concerning that these patients may remain unvaccinated throughout their lifetime despite the possibility of being exposed to vaccine-preventable strains of HPV in the future. A recent (2018) clinical trial assessed HPV vaccine efficacy in HIV-infected MSM who were 27 years old or older ^6^. The study was stopped early because of low vaccine efficacy in preventing anal HSILs ^6^. Further studies are needed to determine whether any PLWH who are 27 years old or older can derive a benefit from vaccination. Unfortunately, the use of therapeutic vaccinations to boost immunity in those already exposed to HPV, including patients with warts refractory to other treatments, is not US Food and Drug Administration (FDA)-approved; however, the successful use of this practice has been reported ^7^.

Acquired epidermodysplasia verruciformis (EV) is a separate clinical condition related to specific subtypes of HPV, namely HPV 5, that is more common in PLWH. It classically manifests with numerous hypopigmented macules and thin papules in sun-exposed areas and often fails to improve after initiation of HAART ^8^. Persons with vertically acquired HIV infection may be more prone to developing EV than persons infected as adults and this is perhaps because their cell-mediated immune system is impaired prior to their first exposure to HPV ^9^. Although the genetic form of EV is associated with an increased risk of developing SCC, the risk of developing SCC in those with acquired EV is not well established in the literature.

### Varicella zoster virus

Varicella zoster virus (VZV) reactivation is more likely to occur in immunocompromised hosts, and recurrence is more likely to be associated with dissemination (more than 10 lesions outside the primary dermatome) or unusual morphologies or both ^10^. Given that VZV reactivation is rare in young persons, we recommend obtaining an HIV test in anybody younger than 50 years of age with VZV reactivation. Providers should also recognize that VZV reactivation may be a sign of immune reconstitution inflammatory syndrome after HAART initiation.

The live vaccine Zostavax was the predominant form of prophylaxis against VZV reactivation for many years. Given the risks associated with live vaccines in immunocompromised hosts, it was not recommended for PLWH. A new recombinant vaccine, Shingrix, is now available. The CDC recommends two doses of Shingrix separated by 2 to 6 months for all patients who are 50 years old or older, including PLWH ^11^. This is true regardless of whether Zostavax was previously administered ^11^.

### Herpes simplex virus

Infection with herpes simplex virus (HSV) 1 and 2 is common throughout the world, but in PLWH, co-infection with HSV may be associated with more frequent and prolonged outbreaks ^12^. Persons with HSV-2 infection are also two to three times as likely to transmit HIV ^13^. There is growing concern about the rise of acyclovir-resistant HSV in immunocompromised hosts, and rates are as high as 10% ^12^. Some have suggested that this is the result of widespread use of suppressive acyclovir among these patients.

Treatment-resistant HSV may present as chronic non-healing ulcerations or lesions (or both) with unusual morphologies, such as verrucous HSV or herpes vegetans ^14, 15^. Second-line treatments for acyclovir-resistant HSV include intravenous foscarnet (which carries a risk of cardiotoxicity) or intralesional cidofovir injections or both ^14, 15^. A new agent, pritelivir, which functions as an HSV helicase-primase inhibitor, has also shown promise. The results of a phase 2 randomized controlled trial (RCT) comparing daily oral pritelivir with oral valacyclovir demonstrated reduced viral shedding and total number of days with genital lesions in the pritelivir group compared with the valacyclovir group ^16^. We advise referral to dermatology for treatment-refractory HSV.

So far, the results of HSV vaccine trials have been disappointing. Two large RCTs of a recombinant glycoprotein vaccine found that vaccines triggered high levels of neutralizing antibodies but ultimately had no efficacy in preventing acquisition of HSV-2 ^17^. Part of the problem is that sustained protection requires more than just high titers of specific neutralizing antibodies; the innate immune system on mucosal surfaces also plays an important role in the early host response. Another approach is to treat known latent HSV infection with vaccinations that boost immunity in order to limit recurrences, more akin to the strategy used with VZV. This has demonstrated some success but is not yet widely employed ^18^. Although numerous HSV vaccinations, including some that may work through mucosal delivery mechanisms, are in the pipeline, no FDA-approved HSV vaccination is currently available ^13, 19, 20^, and two of the three companies that were conducting clinical trials of an HSV vaccination in 2017 recently announced that they are no longer actively pursuing this ^21^.

### Syphilis

Over the last decade, rates of syphilis have been rising ^22^. Some have theorized that this may be an unintended consequence of HIV pre-exposure prophylaxis (PrEP) because MSM who initiate PrEP may reduce the use of strategies to prevent other sexually transmitted infections (STIs). In a 2016 meta-analysis, investigators found that MSM using PrEP were 44.6 times more likely to acquire syphilis compared with those who were not using PrEP ^23^. Two recent trials have explored this further. In a prospective, open-label study of 328 MSM in Amsterdam (2018), investigators found that, after 6 months of PrEP use, the number of receptive and insertive condomless anal sex acts rose from 11 to 14 but that the number of sex partners remained the same and the prevalence of STIs did not change ^24^. In a second open-label prospective study published in 2017, PrEP was associated with reduced use of condoms, but the incidence of STIs also did not rise during the 18-month follow-up period ^25^.

All patients initiating PrEP should be counseled extensively about techniques for preventing other STIs. We recommend confirming a syphilis diagnosis with laboratory tests or skin biopsy (or both), treating empirically if confirmatory tests are unavailable, and monitoring rapid plasma reagin (RPR) at frequent intervals after treatment to detect for re-infection or treatment failure or both. Any evidence of neurosyphilis, including signs of ocular syphilis, merits workup with a lumbar puncture. When diagnosed, neurosyphilis requires treatment with intravenous penicillin G for 10 to 14 days ^26^.

In addition, congenital syphilis has been on the rise in the US ^27^. The CDC recommends testing all women for syphilis by using an RPR early in pregnancy to prevent the numerous permanent sequelae of congenital syphilis. Furthermore, all patients at high risk for acquiring syphilis—including those with a history of syphilis, incarcerated persons, drug users, those with multiple or concurrent sexual partners, those living in high-prevalence areas, and those with other STIs—should be tested with repeat RPR in the third trimester ^27^.

## HIV and inflammatory skin disease

### Psoriasis

Psoriasis is a relatively common, chronic inflammatory skin condition affecting about 2 to 3% of the world population ^28^. Recent data suggest that the prevalence of psoriasis may be as high as 5.4% in PLWH ^29^. In this population, psoriasis may occur *de novo* or as a flare in the setting of a history of the skin disease. Moreover, the clinical course of both cutaneous psoriasis and psoriatic arthritis tends to be more severe and refractory in HIV-infected individuals than in the general population ^30^. As such, treatment with topical corticosteroids or phototherapy alone (or both) may be insufficient. Given that second- and third-line management strategies for psoriasis tend to involve immunosuppressive agents, dermatologists have historically been uncomfortable escalating therapy in patients with an underlying HIV infection. Although an RCT investigating the use of immunosuppressive agents for psoriasis in this population has yet to be conducted, a recent review highlighted 25 cases of systemic therapy being used in this setting ^31^. Biologic agents—specifically tumor necrosis factor-alpha inhibitors such as etanercept, infliximab, and adalimumab—have been used successfully and safely to clear multiple cases of psoriasis, and their benefits have been sustained after several months of treatment ^31^. There was only one report of a serious infection occurring in a patient who was concomitantly controlled on HAART ^31^.

We recommend that patients with newly diagnosed HIV/AIDS first be given an opportunity to respond to treatment with anti-retroviral therapies given that restoration of the immune system is often associated with improvement in skin disease ^32^. In the interim, topical therapies, phototherapy, or systemic retinoids such as acitretin (or a combination of these) may be employed in an attempt to achieve disease control. We further suggest that, in patients whose skin disease is refractory to these interventions and in settings where regular follow-up is available to monitor for symptoms and signs of infection, severe and refractory psoriasis be treated with all available therapies, including biologic agents.

### Atopic dermatitis and other pruritic disorders

Dry skin, atopic dermatitis, prurigo nodularis (PN), pruritic papular eruption (PPE), and idiopathic pruritus have long plagued PLWH, in whom the prevalence of these conditions is as high as 37.5% ^33– 35^. In a recent study, African-American patients with PN were 10.5 times more likely to have HIV infection than race-matched controls with atopic dermatitis ^33^. Moreover, the evidence suggests that the degree of dry, itchy skin reflects the overall burden of immunosuppression; lower CD4 ^+^ T-cell counts have been associated with higher rates of pruritus and atopy ^29, 34^. In the last decade, the nature of the relationship between HIV infection and chronically dry or itchy skin (or both) has been further elucidated. HIV infection provokes a T helper 2 (Th2)-predominant immunophenotype, similar to that seen in patients with intrinsic atopic dermatitis and other allergic conditions ^36^. This cytokine profile in turn can disrupt the skin barrier, even in patients with no history of atopy ^37, 38^. The skin of PLWH has been shown to have a lower epidermal lipid content, partially accounting for this defective skin barrier ^38^. Interestingly, this is similar to the findings seen as a result of normal aging in non-HIV-infected elderly persons, who also commonly have chronic pruritus ^38^. Patients with a previously low CD4 ^+^ T-cell count nadir (<150 cells) have been found to have significantly drier skin ^37^ and may fail to completely recover from their pruritic conditions even after anti-retroviral drugs have restored their CD4 ^+^ T-cell counts and made viral loads (VLs) undetectable. PPE, a condition seen most commonly in PLWH living in low- and middle-income countries, is thought to be driven by an exaggerated immune response to arthropod bites in this population, again due to hyperactive Th2-driven immune pathways ^39^. Given the high prevalence of PN and atopy in PLWH, we recommend testing for HIV in patients with intractable itch or newly diagnosed PN.

## HIV and skin cancer

### Non-melanoma skin cancer

Non-melanoma skin cancers (NMSCs)—basal cell carcinomas (BCCs) and SCCs—are the most prevalent cancer type in the US ^40^. In most patients, these are the result of chronic skin damage induced by ultraviolet (UV) radiation. This risk factor can be especially problematic for communities that have historically valued the aesthetic of tanned skin, including the homosexual male population.

Our understanding of the relationship between HIV-induced immunosuppression and NMSC is evolving. An early study found no relationship between HIV and NMSC compared with age-matched immunocompetent controls, and investigators concluded that the development of NMSCs seemed to be determined by the same genetic and environmental factors that trigger skin cancers in immunocompetent individuals ^41^. However, given that the average age of patients in this study was the mid-40s, reflecting the average age of a PLWH at that time, patients may not have had sufficient time to develop skin cancers, which tend to affect patients much later in life.

More recently, multiple investigations have demonstrated an increased rate of NMSCs among PLWH ^42– 45^. In 2013, physicians at Kaiser Permanente Northern California reported a twofold higher incidence of NMSCs compared with HIV-negative patients ^42^. In 2017, Asgari *et al*. reported that HIV-infected non-Hispanic whites with a history of NMSC were found to be at a higher risk for subsequent new SCCs but not BCCs and also demonstrated a dose-response relationship between this risk and lower CD4 counts as well as higher VLs ^43^. Whereas the overall risk of NMSC was increased by 15% in the HIV-positive population, those with HIV infection and recent biomarkers of severe immune deficiency (CD4 count of less than 200 cells/mL) were at 44% increased risk of a subsequent NMSC and at 222% increased risk of developing SCC in particular ^43^. In a 2018 Danish nationwide cohort study of 4,280 PLWH, HIV conferred an increased risk of both BCC and SCC, and relative risks were 1.79 (95% confidence interval [CI] 1.42–2.22) and 5.40 (95% CI 3.07–9.52), respectively ^44^. A low CD4 ^+^ T-cell count nadir conferred an increased risk of developing SCC ^44^.

The relative risk of developing NMSC among PLWH is not as high as that in solid organ transplant recipients (SOTRs). Several theories attempt to explain why these types of immunosuppression differ so greatly in their tendency to promote cancer growth. HIV seems to promote virally mediated cutaneous malignancies but may play less of a role in promoting UV-induced malignancies. This may be because iatrogenic immunosuppression in SOTRs not only compromises cancer surveillance by the immune system but also impairs cellular repair mechanisms in UV-damaged cells and promotes oxidative DNA damage ^46^. The hypothesis that the relatively higher rate of SCC (relative to BCC) among PLWH could be related to underlying co-infection with the oncovirus HPV, known to drive mucosal SCCs, has been investigated. Although HPV DNA is indeed present in some cutaneous SCCs, transcriptome sequencing failed to identify HPV RNA expression (an indicator of its role in pathogenesis) in cutaneous SCCs ^47^. In contrast, oncogenic HPV 16 and 18 mRNA transcripts were readily identified in mucosal SCCs ^47^. Whether HPV may act as a co-carcinogen with other factors remains controversial ^48^.

We recommend counseling PLWH to protect their skin via sun avoidance, protective clothing, and regular application of sunscreen (sun protection factor of 30 or more). Patients with a history of severe immunosuppression (CD4 count of less than 200 cells/mL) or those with prior NMSCs (or both) should be seen by a dermatologist at least annually for a full body skin check.

### Melanoma

The data are mixed regarding whether HIV infection is linked to a higher incidence of melanoma. In one recent epidemiologic study of patients living in the US or Canada, melanoma incidence was not found to be higher in PLWH ^49^. Conversely, in another recent study, PLWH were significantly more likely than non-HIV-infected persons to be diagnosed with advanced-stage melanoma ^50^. Although the understanding of the role of T-cell immunity in combatting melanoma has improved significantly in recent years, as evidenced by the efficacy of immunomodulatory therapy in treating this condition, it is still unclear whether HIV-induced immune dysfunction plays a significant role in melanoma pathogenesis.

### Kaposi sarcoma

KS, a vascular malignancy mediated by the oncovirus human herpesvirus 8 (HHV-8), continues to be a significant problem for PLWH. In the early HAART era, the rates of KS declined significantly (about 60 to 70%) compared with pre-HAART numbers ^51^. Although the KS incidence continued to decline by about 6% annually from 2000 to 2010 and experts at the National Cancer Institute anticipate a fourfold decline in incidence between 2010 and 2030 ^52^, the HIV-infected population remains at an 800-fold elevated risk of KS compared with the general population ^51^. Recently, investigators found that ongoing HIV exposure, measured as cumulative VL, may promote earlier phases of KS development, independent of CD4 ^+^ T-cell counts ^53^. Given the many consequences of prolonged HIV infection without treatment, we now recommend HAART initiation immediately after HIV diagnosis, regardless of CD4 count.

There have been several reports during the last decade of KS developing in HIV-negative MSM ^54– 56^. This likely reflects the higher rate of HHV-8 infection in this cohort; whereas the seroprevalence is less than 5% in the general US population, it is as high as 20 to 30% in HIV-uninfected MSM ^57^. Likewise, given that the risk of HHV-8 transmission is highest during penetrative anal intercourse, KS incidence is much lower in HIV-positive females ^58^. Suspicious lesions—violaceous patches, plaques, or nodules—in HIV-negative MSM warrant further evaluation with skin biopsy. Unlike KS in HIV-positive patients, KS in HIV-negative MSM tends to have a more indolent course, similar to that of classic KS ^56^.

KS in the developed world continues to be associated with relatively low mortality and is largely manageable with HAART and chemotherapies. Conversely, in sub-Saharan Africa, KS continues to be one of the most common cancers among PLWH and is more likely to be fatal ^59^. This relates to both a genetic predisposition to developing KS—before the HIV epidemic, KS was endemic here—and inadequate access to effective chemotherapies and delay in seeking care because of the stigma associated with HIV and cancer. In some instances, seeking care through alternate practitioners like traditional healers may delay access to conventional care. Health officials in sub-Saharan Africa may require support from both large-scale government interventions and pharmaceutical companies in order to better address this disease burden. However, educating patients and providers is the best option to ensure early diagnosis and an overall better prognosis.

### Merkel cell carcinoma

An oncovirus that is less well-known outside the dermatology world is the Merkel cell polyoma virus (MCV), which plays a role in the development of Merkel cell carcinoma (MCC). MCC is a particularly aggressive and potentially fatal neuroendocrine tumor of the skin and is characterized by high rates of early metastases and local recurrence after surgical excision ^60^. The lesions tend to appear as painless and rapidly growing erythematous skin nodules. The MCV, first identified as an etiologic factor for MCC in 2008, has been found in up to 80% of MCC tumors ^60^. This finding was somewhat surprising because, whereas MCC is rare, the MCV is ubiquitous among human populations, found in about 60 to 80% of persons ^60^. In PLWH, impaired immunity to viral oncogenesis may account for the increased risk of developing MCC, which has been measured as being up to 13.4 times greater in PLWH compared with the general population ^61^. Indeed, MCV DNA has been identified in greater numbers in HIV-infected men compared with healthy controls (59% versus 49%) and is found in greater numbers in those with poorly controlled HIV ^62^. Practitioners caring for PLWH should be aware of this increased risk and inquire about growing skin lesions as part of routine health maintenance.

## Conclusions

HIV continues to be associated with a multitude of infectious, inflammatory, and neoplastic skin manifestations. The morbidity and mortality associated with these conditions have improved as a result of new targeted therapies and vaccines, but much work remains to be done. We recommend sun protection and regular full body skin checks by dermatologists for patients with HIV, the use of available vaccines when possible to limit the risk of developing other viral infections, and consultation with a dermatologist for any skin conditions that are failing to respond to first-line therapies.
